# Effect of *MUC8* on Airway Inflammation: A Friend or a Foe?

**DOI:** 10.3390/jcm7020026

**Published:** 2018-02-06

**Authors:** Hee-Jae Cha, Kyoung Seob Song

**Affiliations:** 1 Department of Parasitology and Genetics, Kosin University College of Medicine, 34 Amnam-dong, Seo-gu, Busan 49267, Korea; hcha@kosin.ac.kr; 2 Department of Physiology, Kosin University College of Medicine, 34 Amnam-dong, Seo-gu, Busan 49267, Korea

**Keywords:** *MUC8*, airway inflammation, inflammatory cytokines, mucus hypersecretion/overproduction, negative regulator

## Abstract

In this review, we compile identifying molecular mechanisms of *MUC8* gene expression and studies characterizing the physiological functions of *MUC8* in the airway and analyzing how altered *MUC8* gene expression in the lung is affected by negative regulators.

## 1. Introduction

In the respiratory tract, mucus consists of lipids, proteins, and an aqueous solution of glycoproteins called mucin [1]. This respiratory mucus is secreted from the intracellular granules of the mucus secretory cells in the superficial airway epithelium and the submucosal glands of the respiratory tract. The synthesis and secretion of respiratory mucus are complementary but regulated by different mechanisms. Respiratory mucus exists as a liquid bilayer structure that consists of an upper gel layer and a lower watery sol layer. The upper layer is moved by the cilia of respiratory epithelial ciliated cells and serves to trap particles and pathogens from inhaled air. The watery sol layer serves as a lubricant to the cilia and allows the mucous layer to spread evenly across the respiratory epithelial cells [1,2]. In addition, Button et al. reported the Gel-on-Brush model to hypothesize that the two different layers’ mucus clearance system stabilized by inhibiting mucus from entering the interciliary space [3]. This model indicated that the distribution of water between the two layers was regulated by this system. An alternative organization has been proposed by Button et al., whereby airway surfaces are lined by two layers: the periciliary layer composed of tethered mucins and the top mucus layer composed of secreted mucins. The success of airway mucus clearance relies on the distribution of water and mucin between these two intercommunicating layers [3].

Mucin proteins are divided into membrane-bound mucins and secreted mucins. Membrane-bound mucins bind to pathogens or are involved in intercellular junctions, and secreted mucins play an important role in the viscoelasticity of the mucous layer [4,5]. *MUC* proteins are encoded by the *MUC* gene in goblet cells or submucosal glands [1]. More than 20 *MUC* genes have been identified to date, 18 of which have been shown to be expressed in the airway (*MUC1*, *MUC2*, *MUC4*, *MUC5AC* , *MUC5B*, *MUC6*, *MUC7*, *MUC8*, *MUC9*, *MUC11*, *MUC12*, *MUC13*, *MUC15*, *MUC16*, *MUC18*, and *MUC20* genes are expressed in normal human nasal epithelial (NHNE) cells, but *MUC3*, *MUC17*, and *MUC19* genes are not; our unpublished data). Many studies report that mucus overproduction and hypersecretion are frequently observed in a number of respiratory diseases, including rhinitis, sinusitis, asthma, chronic obstructive pulmonary disease, and cystic fibrosis [6]. However, the exact physiological roles of *MUC8* protein in respiratory disease remain poorly identified.

## 2.*MUC8*Sequence

**Figure 1:**
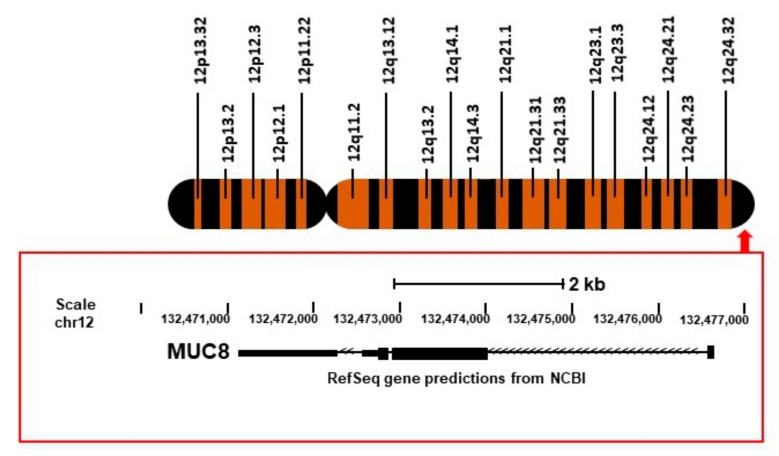
Structure of *MUC8* genes in chromosome 12, searched with the UCSC genome browser (http://genome.ucsc.edu/).

In 1994, the *MUC8* cDNA sequence with a novel tandem repetitive sequence was first partially described by Shankar et al. [7]. Additional new sequences derived by the 3′-rapid amplification of cDNA ends technique were also identified by the same group [8]. The *MUC8* gene is located on the 12q24.3 chromosome and encodes a stop codon, 3′-UTR of 458 bp, a polyadenylation signal, and a poly A+ tail that represents the extreme carboxy terminus of *MUC8* (Figure 1). Based on these sequences, many scientific analyses were conducted by designing the primers for RT-PCR (reverse transcription polymerase chain reaction), peptides for an antibody, and the probes for fluorescence in situ hybridization (FISH). However, the full-length cDNA sequence for *MUC8* has not yet been determined. Because the N- and C-terminals of *MUC8* contain many cysteines and a central region consisting of multiple tandem repeats (rich in serine or threonine), the specific primers could not recognize specific locations (data not shown; our unpublished result). When the primer walking method was performed, we could not read anything other than known sequences.

## 3.*MUC8*Expression

*MUC8* gene expression has been reported to be up-regulated in the sinuses of patients with chronic rhinosinusitis [9]. The expression of *MUC8* at the mRNA level was significantly up-regulated in chronic rhinosinusitis compared with that in normal maxillary sinus mucosa [9]. *MUC8* protein levels were also increased in sinuses with chronic rhinosinusitis compared to normal sinus mucosa, suggesting that *MUC8* may play an important role in the pathogenesis of sinus hypersecretion in chronic rhinosinusitis. Another report showed that mucin secretion, including *MUC8*, was elevated in the lungs of patients with cystic fibrosis but independent of Cl^−^ secretion [10]. In addition, *MUC8* has been reported to be over-expressed in reactive oxygen species-induced chronic airway inflammation [11]. According to these studies, the expression patterns of the *MUC8* gene are typically associated with inflammation. However, the detailed mechanisms showing whether the *MUC8* gene increases or alleviates inflammation in the airway microenvironment are still not clear. Even though scientists have discovered the relationship between *MUC8* expression in the proliferation and differentiation of tracheobronchial cells or mucus hypersecretion in human airway epithelial cells [12], *MUC8* expression is higher than *MUC5AC* expression in nasal polyp biopsies [13]. Importantly, the physiological functions of *MUC8* are still unknown in spite of expression in the airway.

## 4.*MUC8*Function and Signaling

*MUC8* is suggested to play an important role in respiratory disease and inflammatory responses; however, there are many obstacles to studying the function of *MUC8*. The full-length cDNA sequence for *MUC8* has not yet been determined, nor have its functions been fully characterized. Furthermore, the murine *MUC8* homolog has not yet been identified, which has greatly hindered the generation of genetically-modified *MUC8* mouse lines. In order to determine the physiological function of *MUC8* in airway disease, we silenced the *MUC8* gene using a small-interfering RNA (siRNA) in human airway epithelial cells [14]. The ATP/P2Y_2_ complex actively induces airway inflammation by stimulating IL-1α and IL-6. Moreover, ATP increased P2Y_2_-mediated upregulation of *MUC8* expression. However, treatment of airway cells with *MUC8* siRNA stimulated ATP/P2Y_2_-mediated upregulation of IL-1α and IL-6, whereas TGF-β and IL-1 receptor antagonist were reduced. In contrast, siRNA-mediated silencing of *MUC8* downregulated the production of inflammatory chemokines that were increased by ATP-mediated signaling and also ablated ATP/P2Y_2_-mediated chemotaxis. Recently, the importance of mitogen-activated protein kinases (MAPKs) was reported to increase the expression of *MUC8* gene [15,16,17,18,19,20,21]. Interestingly, Asian sand dust induced *MUC8* expression via toll-like receptor (TLR) 4-mediated MAPK activation [22]. MAPK pathways are considered to be most important in transferring inflammatory signals from the cell surface to the nucleus [23,24]. The MAPK pathway is related to cell proliferation, differentiation, apoptosis, cytoskeletal remodeling, and the cell cycle. Interestingly, cAMP-responding element-binding (CREB) protein could bind to c-Ets1 to regulate ATP-dependent *MUC5AC* gene expression [6], and the protein interaction between Suppressor of Cytokine Signaling (SOCS) 3 and Non-POU domain-containing, octamer-binding protein (NonO) inhibited IL-1β-induced *MUC8* gene expression, suggesting that intracelluar protien could act as a negative regulator to maintain homeostasis during airway inflammation [25]. However, the mechanism of *MUC8* gene expression during inflammation in normal airway epithelial cells has not yet been demonstrated, because the signal transduction mechanism is heavily dependent on stimulant and cells.

## 5. Conclusions

**Figure 2:**
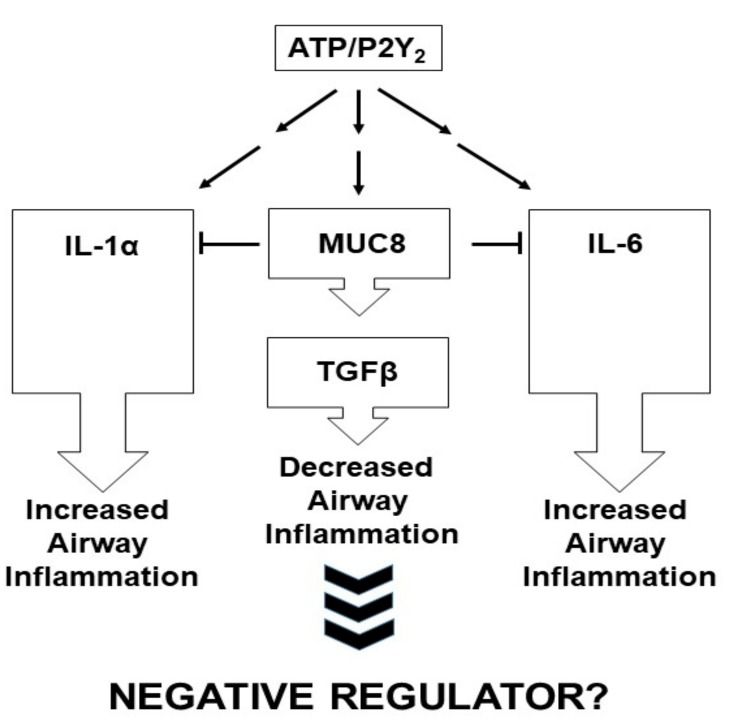
Schematic diagram of the regulation of ATP/P2Y_2_-induced airway inflammation by *MUC8*. *MUC8* may inhibit the ATP/P2Y_2_-mediated upregulation of IL-1α and IL-6, whereas it induces the activity of TGF-β and IL-1 receptor antagonist.

Taken together, these results suggest that *MUC8* may function as an anti-inflammatory mucin that participates in the inflammatory response by reducing the ATP/P2Y_2_-mediated activation of IL-1α and IL-6 (Figure 2). However, many more studies are required to establish that *MUC8* transcripts induce anti-inflammatory responses. As the entire nucleotide sequence of *MUC8* is not determined, it is impossible to produce *MUC8* recombinant proteins or overexpression vectors to observe whether they directly affect inflammatory and anti-inflammatory cytokine expression. Production of knock-out or transgenic mice for *MUC8* is also needed to define the functional role of *MUC8* in vivo. In addition, transcriptome or proteome analyses, by silencing the *MUC8* gene to identify its cytokine expression profile, will help to clarify the role of *MUC8* in the airway inflammatory response.

## References

[1] Rogers D.F. (2007). Physiology of airway mucus secretion and pathophysiology of hypersecretion. Respir. Care.

[2] Evans C.M., Kim K., Tuvim M.J., Dickey B.F. (2009). Mucus hypersecretion in asthma: Causes and effects. Curr. Opin. Pulm. Med..

[3] Button B., Cai L.H., Ehre C., Kesimer M., Hill D.B., Sheehan J.K., Boucher R.C., Rubinstein M. (2012). A periciliary brush promotes the lung health by separating the mucus layer from airway epithelia. Science.

[4] Evans C.M., Koo J.S. (2009). Airway mucus: The good, the bad, the sticky. Pharmacol. Ther..

[5] Rose M.C., Voynow J.A. (2006). Respiratory tract mucin genes and mucin glycoproteins in health and disease. Physiol. Rev..

[6] Song K.S., Lee T.J., Kim K., Chung K.C., Yoon J.H. (2008). cAMP-responding element-binding protein and c-Ets1 interact in the regulation of ATP-dependent *MUC5AC* gene expression. J. Biol. Chem..

[7] Shankar V., Gilmore M.S., Elkins R.C., Sachdev G.P. (1994). A novel human airway mucin cdna encodes a protein with unique tandem-repeat organization. Biochem. J..

[8] Shankar V., Pichan P., Eddy R.L., Tonk V., Nowak N., Sait S.N., Shows T.B., Schultz R.E., Gotway G., Elkins R.C. (1997). Chromosomal localization of a human mucin gene (muc8) and cloning of the cdna corresponding to the carboxy terminus. Am. J. Respir. Cell Mol. Biol..

[9] Lee H.M., Kim D.H., Kim J.M., Lee S.H., Hwang S.J. (2004). Muc8 mucin gene up-regulation in chronic rhinosinusitis. Ann. Otol. Rhinol. Laryngol..

[10] Finkbeiner W.E., Zlock L.T., Morikawa M., Lao A.Y., Dasari V., Widdicombe J.H. (2011). Cystic fibrosis and the relationship between mucin and chloride secretion by cultures of human airway gland mucous cells. American journal of physiology. Lung Cell. Mol. Physiol..

[11] Kim H.J., Kim C.H., Ryu J.H., Joo J.H., Lee S.N., Kim M.J., Lee J.G., Bae Y.S., Yoon J.H. (2011). Crosstalk between platelet-derived growth factor-induced nox4 activation and muc8 gene overexpression in human airway epithelial cells. Free Radic. Biol. Med..

[12] Gray T., Koo J.S., Nettesheim P. (2001). Regulation of mucous differentiation and mucin gene expression in the tracheobronchial epithelium. Toxicology.

[13] Seong J.K., Koo J.S., Lee W.J., Kim H.N., Park J.Y., Song K.S., Hong J.H., Yoon J.H. (2002). Upregulation of *MUC8* and downregulation of *MUC5AC* by inflammatory mediators in human nasal polyps and cultured nasal epithelium. Acta Otolaryngol..

[14] Cha H.J., Jung M.S., Ahn D.W., Choi J.K., Ock M.S., Kim K.S., Yoon J.H., Song E.J., Song K.S. (2015). Silencing of muc8 by siRNA increases P2Y(2)-induced airway inflammation. Am. J. Physiol. Lung Cell. Mol. Physiol..

[15] Lee J.W., Kim Y.I., Im C.N., Kim S.W., Kim S.J., Min S., Joo Y.H., Yim S.V., Chung N. (2017). Grape Seed Proanthocyanidin Inhibits Mucin Synthesis and Viral Replication by Suppression of AP-1 and NF-κB via p38 MAPKs/JNK Signaling Pathways in Respiratory Syncytial Virus-Infected A549 Cells. J. Agric. Food Chem..

[16] Song S.Y., Bae C.H., Choi Y.S., Kim Y.D. (2016). Cadmium induces mucin 8 expression via Toll-like receptor 4-mediated extracellular signal related kinase 1/2 and p38 mitogen-activated protein kinase in human airway epithelial cells. Int. Forum Allergy Rhinol..

[17] Bae C.H., Jeon B.S., Choi Y.S., Song S.Y., Kim Y.D. (2014). Delphinidin Inhibits LPS-Induced *MUC8* and *MUC5B* Expression Through Toll-like Receptor 4-Mediated ERK1/2 and p38 MAPK in Human Airway Epithelial Cells. Clin. Exp. Otorhinolaryngol..

[18] Song S.Y., Jung E.C., Bae C.H., Choi Y.S., Kim Y.D. (2014). Visfatin induces *MUC8* and *MUC5B* expression via p38 MAPK/ROS/NF-κB in human airway epithelial cells. J. Biomed. Sci..

[19] Bae C.H., Kim J.S., Song S.Y., Kim Y.W., Park S.Y., Kim Y.D. (2013). Insulin-like growth factor-1 induces *MUC8* and *MUC5B* expression via ERK1 and p38 MAPK in human airway epithelial cells. Biochem. Biophys. Res. Commun..

[20] Cho K.N., Choi J.Y., Kim C.H., Baek S.J., Chung K.C., Moon U.Y., Kim K.S., Lee W.J., Koo J.S., Yoon J.H. (2005). Prostaglandin E2 induces *MUC8* gene expression via a mechanism involving ERK MAPK/RSK1/cAMP response element binding protein activation in human airway epithelial cells. J. Biol. Chem..

[21] Moon U.Y., Kim C.H., Choi J.Y., Kim Y.J., Choi Y.H., Yoon H.G., Kim H., Yoon J.H. (2010). AP2alpha is essential for *MUC8* gene expression in human airway epithelial cells. J. Cell. Biochem..

[22] Choi Y.S., Bae C.H., Song S.Y., Kim Y.D. (2015). Asian sand dust increases *MUC8* and *MUC5B* expressions via TLR4-dependent ERK2 and p38 MAPK in human airway epithelial cells. Am. J. Rhinol. Allergy.

[23] Garrington T.P., Johnson G.L. (1999). Organization and regulation of mitogen-activated protein kinase signaling pathways. Curr. Opin. Cell Biol..

[24] Song K.S., Lee W.J., Chung K.C., Koo J.S., Yang E.J., Choi J.Y., Yoon J.H. (2003). Interleukin-1 beta and tumor necrosis factor-alpha induce *MUC5AC* overexpression through a mechanism involving ERK/p38 mitogen-activated protein kinases-MSK1-CREB activation in human airway epithelial cells. J. Biol. Chem..

[25] Song K.S., Kim K., Chung K.C., Seol J.H., Yoon J.H. (2008). Interaction of SOCS3 with NonO attenuates IL-1beta-dependent*MUC8*gene expression. Biochem. Biophys. Res. Commun..

